# Ideas to Assist the Underprivileged Dispossessed Individuals

**Published:** 2012

**Authors:** Fatemeh Heidary, Reza Gharebaghi

**Affiliations:** 1 School of Medicine, Shahid Beheshti University of Medical Sciences, Tehran, Iran; 2 Editorial Office, Medical Hypothesis, Discovery & Innovation Ophthalmology Journal

**Keywords:** Ophthalmology, Poverty, Blindness, Underprivileged dispossessed individuals

**Inequality in health and ophthalmic services**

Unfortunately, the medical and welfare facilities are not homogenously distributed to all parts of the world. In many countries, malnutrition could be considered as the major problem while in many other countries, the side effects of obesity are the causes of mortality and morbidity. Within the sum total of all countries there are huge inequalities as well. 

The same is true in ophthalmic diseases. In many parts of the world, patients have access to the best surgical corneal operations and receive the benefits of the latest methods of minimal invasive surgeries by modern technologists. In many cases, vision improves a few minutes after surgeries, the occuloplastic operations make figures appear younger and more attractive and expensive glasses and high quality intraocular lenses make life more comfortable. However, in other parts of the world many people are deprived of minimal levels of healthcare and ophthalmic services, including the correction of refractive status with simple glasses.

Socioeconomic development and improved living standards in many societies have resulted in greater control of the major communicable causes of blindness, such as trachoma and ocular infectious diseases which has caused significant shifts in the pattern of visual impairment presentation. It has been shown while it is essential to continue to invest efforts in eliminating communicable eye diseases as the cause of visual impairments and blindness, the future challenge lies in the provision of eye care for the effects of non-communicable eye diseases [2].

The relationship between socioeconomic status and various diseases have been well presented in the literature. An inverse association between socioeconomic status and rate of blindness has been reported as well. This is mostly due to diseases that are related with deprivation, such as trachoma and vitamin A deficiency, or through insufficient access to healthcare services, such as cataract removal. It has been established that health care inequalities can be attributed to social factors in many conditions [3]. Many causes of preventable blindness in low-income countries are inarguably associated with poverty [4]. On the other hand, inequalities in healthcare within and between countries present yet another challenge. In some impoverished countries, there is a life expectancy of 48 years and around 20 or more additional years in other countries [5]. As a matter of fact, healthcare and medical services are not distributed equally in most countries. For example, the life expectancy differs between countries or even inside a country because access to health and medical services is not universally available. The same applies to eye care services. Therefore, in addition to improving the availability of healthcare, the decision makers must pay attention to equitable distribution of these services in their countries as well.

**Ideas to assist poor people**

Innovations and discoveries such as vaccination, antibiotics, MRI, and phacoemulsification continuously change the face of healthcare. But what’s next discovery especially in the field of ophthalmology and visual sciences? 

Not long ago, a contest was arranged by British Medical Journal to appreciate outstanding ideas and innovations. The winner of the contest was a designer of low priced glasses that could improve patient’s vision to some extent in low socioeconomic countries. Inventor of the world’s first adjustable glasses has confidence in bringing corrective spectacles to those who need it around £1 without the prerequisite for either ophthalmologists or optometrists. He believes this instrument will have major impacts on quality of life worldwide [1]. BMJ Innovation Expo conference in London and the winner sent a tremendous message to the science community which is that the time has come to focus research on underprivileged and low income Individuals.

Similarly, advancements in healthcare services and access to them should be provided as well. Advancements originate from new ideas and hypotheses. There must be a systemic change to the current system so that the deprived and lower income individuals receive these benefits as well. Our scope is focusing in these kind of ideas.

**Wider perspective in publication**

We are pleased to announce the third issue of Medical Hypothesis, Discovery & Innovation [MEHDI] Ophthalmology Journal which focuses especially on new ideas and important hypotheses in the glaucoma and optic nerve diseases. 

In addition to publishing articles in general ophthalmology and visual sciences, the journal tries to prove feasibility of publishing ideas in subspecialty fields too. When the journal was first being established, some of our colleagues argued that there would be only a few articles submitted to the journal because of its highly specialized focus. With all due respect to our colleagues, we have found that our journal has performed very successfully. Based on the number of manuscripts that have been submitted, it has been demonstrated that there are many capable, enthusiastic and effective researchers in the field of ophthalmology who are happy to have their ideas published in a journal specializing in innovation and ideas.

One of the major areas we are looking to publish papers is in the interdisciplinary studies. As an example, we will include studies that share scope in various experimental sciences with mathematics, computer science, chemistry and visual sciences. Our journal intends to create a forum in which distribution and discussions of different types of research are studied. 

The importance of reform in science and research is essential. Experience has shown that policy makers who are open to new ideas and active listeners of the disenfranchised are more successful. They create the discussions and ideas that lead to growth and evolution. 

Actually, the world is on the threshold of abundant reforms. Scientists should not allow political or economic powers to dominate their respective worlds. The world must be run by scientists’ expertise in a logical and scientific manner. Therefore, our goal is to create an unrestricted environment for the free and open exchange of information. The editors of this journal believe that any idea, no matter how simple, if it has been written in logical and scientific way, could contribute to the progress of science around the world. This journal publishes research articles with innovations, and we welcome manuscripts that address the needs of underprivileged Individuals.

We hereby extend our sincere thanks to the authors, editorial board members, and the staff of the journal. Without their help the journal could not be published continuously. Our journal not only encourages your comments and feedback; it will publish them if they are written scientifically. 

And as a conclusion, our major goal is to encourage the development of ideas that is much needed for attaining future evolutionary goals especially to help the underprivileged dispossessed individuals. 

We all have wishes and ideas to make the world better. We believe there is enormous influence in the sharing of those ideas by distributing in scientific publications. The Future we need is a universal dialogue through a positive vision.

## DISCLOSURE

The authors report no conflicts of interest in this work.
